# Therapeutic delivery of recombinant glucocerebrosidase enzyme-containing extracellular vesicles to human cells from Gaucher disease patients

**DOI:** 10.1186/s13023-024-03376-7

**Published:** 2024-10-02

**Authors:** Keatdamrong Janpipatkul, Nareerat Sutjarit, Amornrat Tangprasittipap, Tai Chaiamarit, Pawarit Innachai, Kanoknetr Suksen, Tanida Chokpanuwat, Thipwimol Tim-Aroon, Usanarat Anurathapan, Natee Jearawiriyapaisarn, Alisa Tubsuwan, Supareak Bowornpinyo, Nithi Asavapanumas, Arthit Chairoungdua, Kanit Bhukhai, Suradej Hongeng

**Affiliations:** 1 Department of Basic Medical Science, Faculty of Medicine Vajira Hospital, Navamindradhiraj University, Bangkok, Thailand; 2 Graduate Program in Nutrition, Faculty of Medicine Ramathibodi Hospital, Mahidol University, Bangkok, Thailand; 3 Office of Research, Academic Affairs and Innovations, Faculty of Medicine Ramathibodi Hospital, Mahidol University, Bangkok, Thailand; 4 Department of Physiology, Faculty of Science, Mahidol University, Bangkok, Thailand; 5 Department of Pediatrics, Faculty of Medicine Ramathibodi Hospital, Mahidol University, Ratchathewi, Thailand; 6 Institute of Molecular Biosciences, Mahidol University, Nakhon Pathom, Thailand; 7 Department of Biotechnology, Faculty of Science, Mahidol University, Bangkok, Thailand; 8 Excellent Center for Drug Discovery, Mahidol University, Bangkok, Thailand; 9 Chakri Naruebodindra Medical Institute, Faculty of Medicine Ramathibodi Hospital, Mahidol University, Bang Pla, Bang Phli, Samut Prakan, Thailand

**Keywords:** Gaucher disease, Glucocerebrosidase, Extracellular vesicles, Macrophages, Neurons, Lentiviral vector, HEK293T cell

## Abstract

**Background:**

Gaucher disease (GD) is one of the most common types of lysosomal storage diseases (LSDs) caused by pathogenic variants of lysosomal β-glucocerebrosidase gene (*GBA1*), resulting in the impairment of Glucocerebrosidase (GCase) enzyme function and the accumulation of a glycolipid substrate, glucosylceramide (GlcCer) within lysosomes. Current therapeutic approaches such as enzyme replacement therapy and substrate reduction therapy cannot fully rescue GD pathologies, especially neurological symptoms. Meanwhile, delivery of lysosomal enzymes to the endocytic compartment of affected human cells is a promising strategy for treating neuropathic LSDs.

**Result:**

Here, we describe a novel approach to restore GCase enzyme in cells from neuropathic GD patients by producing extracellular vesicle (EVs)-containing GCase from cells overexpressing *GBA1* gene. Lentiviral vectors containing modified *GBA1* were introduced into HEK293T cells to produce a stable cell line that provides a sustainable source of functional GCase enzyme. The *GBA1*-overexpressing cells released EV-containing GCase enzyme, that is capable of entering into and localizing in the endocytic compartment of recipient cells, including THP-1 macrophage, SH-SY5Y neuroblastoma, and macrophages and neurons derived from induced pluripotent stem cells (iPSCs) of neuropathic GD patients. Importantly, the recipient cells exhibit higher GCase enzyme activity.

**Conclusion:**

This study presents a promising therapeutic strategy to treat severe types of LSDs. It involves delivering lysosomal enzymes to the endocytic compartment of human cells affected by conditions such as GDs with neurological symptoms, as well as potentially other neurological disorders impacting lysosomes.

**Supplementary Information:**

The online version contains supplementary material available at 10.1186/s13023-024-03376-7.

## Introduction

Gaucher disease (GD) is a prevalent lysosomal storage disorder (LSD) caused by pathogenic variants in the *GBA1* (606463) gene, which encodes the glucocerebrosidase (GCase) enzyme. GCase plays a key role in degrading a metabolic waste substance known as glucosylceramide (GlcCer) within lysosomes. Variation in the *GBA1* gene lead to a significant reduction in GCase activity, resulting in the accumulation of GlcCer in lysosomes, predominantly observed in macrophages [1]. This accumulation could transform macrophages into Gaucher cells that infiltrate various organs including bone marrow, liver, spleen, and bones, consequently causing pancytopenia, hepatomegaly, splenomegaly, and bone abnormalities [2, 3]. GD can be classified into three subtypes based on clinical manifestations. Type 1 GD is the most common form and is primarily characterized by visceral symptoms. While it was previously considered that neurological symptoms were absent, accumulating evidence now suggests an increased risk of Parkinson’s disease in individuals with Type 1 GD [4, 5]. Conversely, type 2 and type 3 GDs are associated with neurological impairments, with severe symptoms in type 2 and variable ones in type 3 [6].

The conventional therapy for GD involves replacing the deficient GCase with the recombinant GCase. Enzyme replacement therapy (ERT) has effectively treated the visceral symptoms of type 1 GD. However, the efficacy of ERT is limited when addressing neurological symptoms in type 2 and 3 GDs due to the inability of recombinant enzymes to cross the blood-brain barrier (BBB) [7]. Consequently, systemically administered enzymes fail to reach the brain parenchyma, leaving GD patients with central nervous system (CNS) involvement without effective treatment. Various strategies have been employed to deliver GCase to neurons effectively, such as linking GCase to different membrane-binding peptides such as the Tat peptide [8], Apolipoprotein B (ApoB), and the rabies glycoprotein-derived peptide (RDP) [9]. Additionally, a fragment of the flexible hinge region from IgA (IgAh) was introduced between RDP and GCase to create a recombinant RDP-IgAh-GCase. This recombinant DNA design exhibits a potential therapeutic option for neuropathic GD due to its efficient neuron-targeting properties and its potential to cross the BBB [9]. Nonetheless, the peptide-linked recombinant GCase has not been tested for therapeutic efficacy in various cell types—including human cells from LSD patients.

Taking a step further, using nanotechnological approaches to enhance the efficiency of recombinant enzyme production and to facilitate their cell-targeting specificity emerges as a promising strategy to develop effective LSD therapies [10]. Nanoparticles, including virus-like nanoparticles (VLPs), have been investigated as potential vehicles for ERT in GD [11, 12]. Recently, extracellular vesicles (EVs), with the size range from 30 to 150 nm, have been employed as delivery vehicles for recombinant proteins [13]. EVs are biologically important mediators of cell-to-cell communication in health and disease, and EVs naturally contain active biomolecules such as proteins, nucleic acids, and lipids from the donor cells. In addition to their biocompatible properties, EVs demonstrate higher in vivo transfection capacity and lower immunogenic responses compared to other non-viral platforms [14]. Numerous studies have explored the potential of EVs and exosomes as drug delivery vehicles capable of crossing or bypassing the BBB [15, 16]. Importantly, EVs can be engineered to enhance targeting efficacy and therapeutic effects using genetically modified cells as producing sources, in which the vehicles and therapeutic proteins are jointly produced. Furthermore, EVs can be loaded chemically and mechanically after production and isolation, enabling the incorporation of nucleic acids, enzymes, and therapeutic proteins. This approach holds particular relevance for treating neuropathic forms of GD, where ERT is insufficient. This study characterizes EVs containing functional GCase from genetically modified cells overexpressing the *GBA1* gene and investigates the delivery of GCase-containing EVs in various types of target cells, including macrophages, neuronal cell lines, and neurons/macrophages from LSD patient-derived induced pluripotent stem cells (iPSCs).

## Materials and methods

### Development of lentiviral vector carrying *GBA1* gene (LV-*GBA1*)

The lentiviral vector included the CMV promoter to drive *GBA1* gene expression in mammalian cells. The vector contained a puromycin resistance gene under the control of the phosphoglycerate kinase (PGK) promoter to facilitate the selection of transduced cells. Production of LV-*GBA1* involved transiently transfected HEK293T cells with the transfer plasmid containing the *GBA1* gene (pLVX-CMV-*GBA1*), along with packaging plasmids pMD2G (VSV-G envelop) and psPAX2 (Gag-Pol, REV, Tat), using Polyethylenimine (PEI, Euromedex). After 16 h of transfection, the transfection medium was replaced by fresh Dulbecco’s modified Eagle medium (DMEM) medium (Thermo Scientific, MA, USA). After 24 h, cell supernatant was collected and filtered through a 0.45 μm Durapore filter (Millipore) to remove cell debris. Lentiviral vectors were concentrated and purified by incubating with Lentivirus Precipitation Solution (Takara Bio USA, Inc.) at 4 °C overnight, followed by centrifugation at 1,500 x g at 4 °C for 1 h. After centrifugation, the viral pellet was resuspended in PBS and divided into aliquots for immediate freezing and kept at -80 °C. The infectious viral titer of the vector was determined using the Lenti-X qRT-PCR Titration Kit (Takara Bio USA, Inc.).

### Establishment of stable HEK293T overexpressing *GBA1* gene (HEK293T-*GBA1*)

HEK293T cells were seeded on a 6-well plate at a density of 1 × 10^5^ cells/well and incubated for 24 h. The cells were then transduced with LV-*GBA1* at a multiplicity of infection (MOI) of 1, and incubated for 48 h. The transduction medium was replaced with regular culture medium and incubated for another 24 h. Subsequently, the transduced cells were exposed to puromycin at a concentration of 2.5 µg/mL for 48 h. The remaining cells were cultured for an additional 10 days. Overexpression of the *GBA1* gene was confirmed by assessing protein levels and enzymatic activity of GCase.

### Cell culture and differentiation

The human embryonic kidney cell line (HEK293T), human monocytic cell line (THP-1), and human neuroblastoma cell line (SH-SY5Y) were obtained from the American Type Culture Collection (ATCC, VA, USA). HEK293T cells were cultured in DMEM medium supplemented with 10% fetal bovine serum (FBS) (Hyclone, UT, USA) and 1% Penicillin-Streptomycin (Pen-strep) solution (100 U/ml penicillin and 100 µg/ml streptomycin) (Thermo Scientific, MA, USA). THP-1 cells were cultured in RPMI 1640 medium (Thermo Scientific, MA, USA) containing 10% FBS, 1% Pen-strep, 1 mM sodium pyruvate, and 0.1 mM 2-mercaptoethanol. SH-SY5Y cells were cultured in DMEM/F12 medium with 10% FBS and 1% Pen-strep. Cells were incubated at 37 °C in a humidified atmosphere with 5% CO2.

#### Differentiation of THP-1 monocytes to macrophages

For macrophage differentiation, THP-1 monocytes were seeded onto 24-well and 6-well plates at a density of 1 × 10^5^ or 1 × 10^6^ cells/mL for immunostaining and enzymatic activity assays, respectively. Differentiation into macrophages was achieved by incubating the cells with culture medium containing 200 nM Phorbol 12-myristate 13-acetate (PMA) (Sigma-Aldrich, MO, USA) for 5 days.

#### Differentiation of SH-SY5Y neuroblast to mature neuron

Differentiation of SH-SY5Y cells into mature neurons was accomplished by seeding them onto a 6-well plate at a density of 5 × 10^5^ cells/well, followed by incubation for 24 h. Subsequently, cells were induced to differentiate into mature neurons by incubating in DMEM/F12 medium containing 1% FBS and 10 µM retinoic acid for 7 days.

#### Generation of induced pluripotent stem cells (iPSCs) from hematopoietic stem cells (HSCs) of neuropathic GD patients

The iPSCs from two patients of type 3 GD [17, 18] and a healthy control [19] were utilized in this study and were cultured according to a previously described protocol using our in-house Essential 8 medium [18].

#### Differentiation of monocyte from human iPSCs

The protocol for macrophage differentiation from human iPSCs was modified from Cui et al., 2021 [20]. Briefly, forty cluster of undifferentiated iPSCs were seeded per well in a matrigel-coated six-well plate to generate around 20 colonies sized 400–700 nm in a week. On Day 0, the maintenance medium was switched to E8 medium supplemented with 80 ng/ml of VEGF (100 − 20, Peprotech), 80 ng/ml of BMP4 (120-05ET, Peprotech), and 4 µM of CHIR99021 (2520691, Peprotech). At Day 2, the medium was replaced with in house-E6 medium supplemented with 80 ng/ml of VEGF, 50 ng/ml of FGF-basic (100-18B, Peprotech), 50 ng/ml of SCF (300-07, Peprotech), and 2 µM of SB431542 (3014193, Peprotech). At Day 4, the cells were served with the basal medium which was Stemline II (S0192, Sigma) with ITS (SCM054, Sigma), supplemented with 40 ng/ml of VEGF, 50 ng/ml of SCF, 10 ng/ml of TPO (300 − 18, Peprotech), 50 ng/ml of IL-3 (200-03, Peprotech), and 50 ng/ml of FLT-3 (300 − 19, Peprotech). At Day 7, a half-medium change was carried out with basal medium supplemented with 50 ng/ml of SCF, 10 ng/ml of TPO, 50 ng/ml of IL-3, 50 ng/ml of FLT-3, and 50 ng/ml of M-CSF (HY-P7050A, MedChemExpress (MEC)). At Day 10–14, a medium as carried out with basic medium supplemented with 50 ng/ml of FLT-3, 50 ng/ml of M-CSF, and 25 ng/ml of GM-CSF (HY-P7016, MEC). The floated cells were harvested for phenotype analysis by flow cytometry.

#### Differentiation of macrophages from iPSCs-derived monocytes

The floated iPSCs-derived monocytes were collected for further differentiated with Roswell Park Memorial Institute (RPMI) 1640 medium (21875034, Gibco) supplemented with 10% FBS (F7524, Sigma) and 50 ng/ml of M-CSF toward to macrophages in 8–11 days. For monitoring the macrophage morphology, cells were collected for cytospin preparation followed by staining with Wright-Giemsa stain.

For macrophage cell surface marker detection, the cells were gathered and suspended in a blocking solution (0.05% BSA in 1X PBS). The cells were stained with an optimal amount of each antibody, including CD34 (343606, BioLegend), CD45 (304016, BioLegend), CD14 (325607, BioLegend), CD163 (333618, BioLegend), and CD80 (305232, BioLegend). Staining was carried out in the dark at 4 °C for 20 min. Subsequently, the cells were washed with 500 µL of 0.05% BSA in 1X PBS and centrifuged at 600 g for 5 min. The stained cell was subjected to BD FACSLyric. Expression levels were analyzed using FlowJo 10.9.0.

#### Differentiation of GD iPSCs to mature neuron

iPSCs from passage 25–35 range were used for the process of neural cell induction. The neural induction medium contained an equal amount of DMEM/F-12 and advanced neurobasal medium (Gibco, USA), supplemented with 1X GlutaMAX (Gibco, USA), 1X B-27 supplement (without Vitamin A) (Gibco, USA), 1X N-2 supplement (Gibco, USA), 10 µM SB431542 (Sigma-Aldrich, USA), 0.1 µM LDN193189, and 0.1X penicillin/streptomycin, to facilitate the differentiation of iPSCs into neural progenitor cells (NPCs). Next, the neural expansion medium containing an equal amount of DMEM/F-12 and advanced neurobasal medium with 1X GlutaMAX, 1X B-27 supplement (without Vitamin A), 1X N2 supplement, 20 ng/µl FGF-2, 20 ng/µl EGF, and 0.1X penicillin/streptomycin was used in order to further cultivate NPCs. The final differentiation stage of mature neurons was achieved on NPCs at passage 4–5 by culturing in a neural differentiation medium, that has an equal amount of DMEM/F-12 and advanced neurobasal medium, 1X GlutaMAX, 1X B-27 supplement (without Vitamin A), 1X N2 supplement, 50 µM DB-cAMP, 200 µM Ascorbic acid, 20 ng/ml BDNF (Peprotech, USA), and 10 ng/ml GDNF (Peprotech, USA).

### EV isolation

Non-transduced HEK293T cells (HEK293T-NT) and stable HEK293T cells overexpressing *GBA1* gene (HEK293T-*GBA1*) were cultured in 75 cm² culture flasks using DMEM medium supplemented with 10% FBS, at 37 °C in a 5% CO2 incubator. Upon reaching 70–80% confluence, cells were cultured with fresh medium containing 10% FBS depleted of EVs (centrifuged at 100,000 × g for 18 h). After 48 h of incubation, culture supernatant was collected. EVs were purified using differential centrifugation. Initially, centrifugation at 1,200×g for 10 min at 4 °C was performed to remove cellular debris. The resulting supernatant was subjected to 10,000×g centrifugation for 30 min to eliminate macroparticles. The supernatant was filtered using a 0.2 μm filter and then centrifuged at 110,000×g for 90 min. EV pellets were washed with PBS, followed by centrifugation at 110,000×g for 75 min. EV pellets were resuspended in 50–100 µL PBS and stored at -80 °C.

### EV characterization

#### Electron microscopy

EVs were applied to formvar-carbon-coated copper grids (Electron Microscopy Sciences, Hatfield, PA), incubated at room temperature for 20 min, and then washed with PBS buffer. The samples were fixed with 1% glutaraldehyde for 5 min and subsequently washed with distilled water. Staining was performed using 2% uranyl acetate for 1 min, and samples were allowed to dry at room temperature before visualization with a transmission electron microscope (Hitachi, Tokyo, Japan).

#### Nanoparticle tracking analysis (NTA)

The analysis of EVs particles’ size distribution was conducted through nanoparticle tracking using the NS300 nanoparticle analyzer (NTA) (NanoSight, Malvern, UK). For our recordings, a camera level of 15 was consistently applied, along with automatic functions for post-acquisition settings, except for the detection threshold. Samples were appropriately diluted in PBS within the range of 1:100 to 1:10,000 to achieve a particle count between 1 × 10^8^ and 1 × 10^9^ per ml. The camera focus was optimized to ensure particle visibility as distinct dots. Employing the script control function, we recorded five 60-second videos for each sample.

#### Western blot analysis

Total protein lysates were extracted from stable HEK293T-NT and HEK293T-*GBA1* cell lines using a lysis buffer containing 50 mM Tris-HCl pH 7.4, 150 mM NaCl, 1 mM EDTA, 1% TritonX-100, 1 mM NaF, 1 mM Na_3_VO_4_, 1 mM PMSF, and a protease inhibitor cocktail. Equal amounts of protein samples or EV samples were mixed with 4X Laemmli buffer and heated at 95 °C for 5 min. Proteins were separated by sodium dodecyl sulfate-polyacrylamide gel electrophoresis (SDS-PAGE) and subsequently transferred to a nitrocellulose membrane by electroblotting. Membranes were incubated overnight at 4 °C with the following primary antibodies: anti-Flotillin-1, anti-TSG101, anti-CD81, anti-Connexin, and anti-β-actin. Afterward, membranes were washed with Tris-buffered saline with Tween 20 (TBST), followed by incubation with HRP-conjugated goat anti-rabbit or HRP-conjugated goat anti-mouse secondary antibodies. Membranes were then given a final wash with TBST. Signals were detected using enhanced SuperSignal West Pico Chemiluminescent Substrate (Thermo Scientific Fisher, Waltham, MA). The following antibodies were utilized: anti-Flotillin-1 (BD Biosciences, San Jose, CA); anti-CD81 (B-11) (Santa Cruz Biotechnology, Dallas, TX); anti-TSG101 (4A10) and anti-Calnexin (22595) (Abcam, Cambridge, UK); and β-actin (Sigma-Aldrich, MO, USA).

### EV uptake, immunofluorescence, and live imaging

Cells were seeded on glass coverslips in 24-well plates and cultured with medium containing 10% EV-depleted FBS at 37 °C in a humidified 5% CO_2_ incubator. For EV uptake, cells were treated with 10 µg of PKH-67 dye-labeled EVs for 2, 6, 12, and 24 h and subsequently analyzed using immunofluorescence imaging. For immunofluorescence, the cells were washed with cold Ca^2+^- and Mg^2+^-containing PBS (PBS^++^), fixed with 100% methanol for 10 min, and then incubated with a permeabilizing/blocking buffer (0.3% Triton X-100, 0.3% BSA, and 1% FBS) for 30 min. Alexa Fluor 594 Phalloidin (Invitrogen, USA) was used to label F-actin. Next, the cells were counterstained with DAPI (Thermo Scientific Fisher, Waltham, MA) for 10 min at room temperature to detect nuclei. The stained cells were washed five times with PBS^++^ and then mounted. Fluorescence signals were visualized at room temperature using LSM900 confocal microscopy (Carl Zeiss, Germany).

For live imaging in iPSC-derived neurons, SiR-Lysosome Kit (SpiroChrome, Switzerland), SPY555-tubulin (SpiroChrome, Switzerland), and Hoechst 33,342 (H3570, Invitrogen, USA) were used to detect lysosomes, microtubules, and nuclei respectively, according to the manufacturer’s instructions. Fluorescence signals were visualized at room temperature using LSM900 confocal microscopy.

### Enzyme activity assay

The enzyme activity assay was performed using the degradation of 4-methylumbelliferyl β-D-glucopyranoside. Cell pellets from the culture were lysed in 100 mM citrate buffer (pH 5.2) containing 0.1% (vol/vol) Triton X-100 using an ultrasonic homogenizer (Omni-Ruptor 4000). The lysates were then assessed for protein concentration using the BCA assay (Thermo Scientific) with BSA as the standard. Subsequently, total proteins of 20 µg were incubated in phosphate-citrate buffer at pH 5.4, in the presence of 4-methylumbelliferyl β-D-glucopyranoside substrates (4-MUG). The 4-MUG will be converted to 4-Methylumbelliferone (4-MU), a fluorescent product. The fluorescent signal (Ex wavelength 360 nm and Em wavelength 450 nm) was measured using a BioTek Cytation 7 cell imaging reader (Agilent Technologies, CA, USA), and the enzyme activity was calculated using a standard curve of concentration-dependent 4- MU fluorescence.

### Statistical analysis

All presented data are expressed as mean ± S.E.M. from triplicate independent experiments. Statistical comparisons between groups were conducted using unpaired student’s *t*-test or one-way ANOVA, followed by a Tukey-Kramer post hoc test. P-values less than 0.05 (*p* < 0.05) were considered statistically significant.

## Results

### Generation of the lentiviral vector carrying a functional GCase and establishment of *GBA1*-overexpressing cell line

**Fig. 1:**
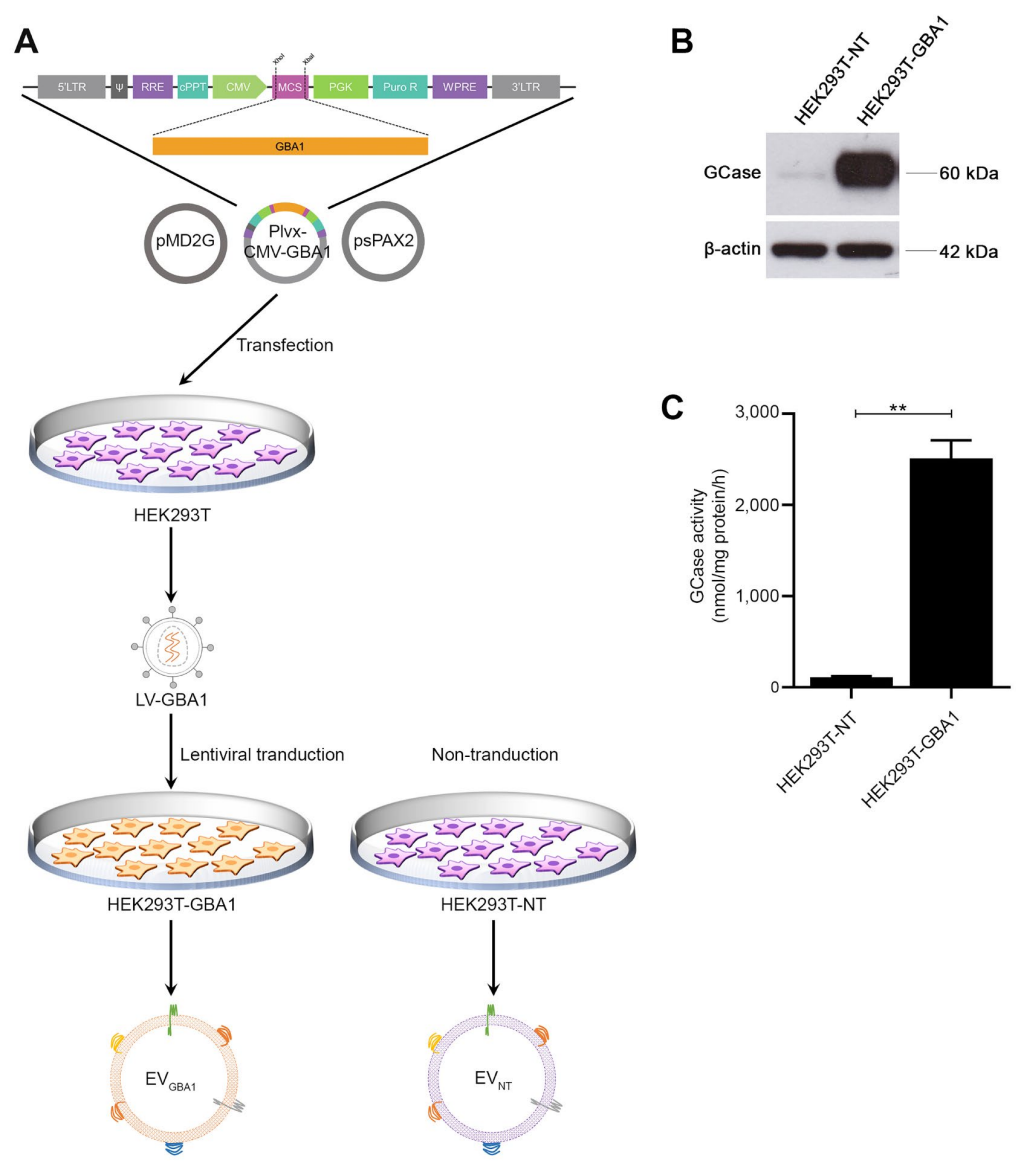
The establishment of *GBA1*-overexpressing cells and isolation of extracellular vesicle. (**A**) The construction of the *GBA1* transfer plasmid used for producing *GBA1* lentiviral vector (LV-*GBA1*) and the transduction of LV-*GBA1* to HEK293T, establishing HEK23T overexpressing *GBA1* that secrete extracellular vesicle containing Gcase enzyme. HEK293T were transduced with LV-*GBA1* at MOI of 1 and treated with 2.5 µg/mL of puromycin to select positive cells containing LV-*GBA1* (**B**) Glucocerebrosidase (Gcase) protein level in HEK293T overexpressing *GBA1* were determined by Western Blot analysis, and (**C**) Gcase enzyme activity in HEK293T overexpressing *GBA1* cells was determined by enzymatic assay. Individual data relative to HEK293T-NT are represented on bar chart with mean ± SEM, *n* = 3. **p < 0.01 compared with HEK293T-NT (unpaired student’s t-test)

The illustration for plasmid construction and the production of *GBA1*-overexpressing HEK293T stable cells (called HEK293T-*GBA1*) was demonstrated in Fig. 1A. The transfer plasmid, designated as the pLVX-CMV-*GBA1* plasmid, was constructed to contain the *GBA1* gene under the control of the CMV promoter. The modified plasmid size was verified using restriction enzymes XhoI and XbaI, producing two distinct fragments of DNA, with sizes of 1,665 bp and 8,043 bp, as shown in clone #2 and #3 (Fig. S1). In this study, clone #3 was selected for lentiviral vector production. The titer of the vector was approximately 1.47 × 10^9^ TU/ml.

To confirm the functionality of HEK293T-*GBA1* cells, both glucocerebrosidase (GCase) protein expression and its enzymatic activity were evaluated through western blot and enzymatic assays, respectively. As expected, we found predominant expression of GCase protein (Fig. 1B) and heightened enzyme activity (Fig. 1C) in the HEK293T-*GBA1* cells, compared to non-transduced cells (HEK293T-NT). These findings strongly indicate the successful generation of a stable cell line harboring functional GCase activity.

### Isolation and characterization of HEK293T stable cell-derived EVs

**Fig. 2:**
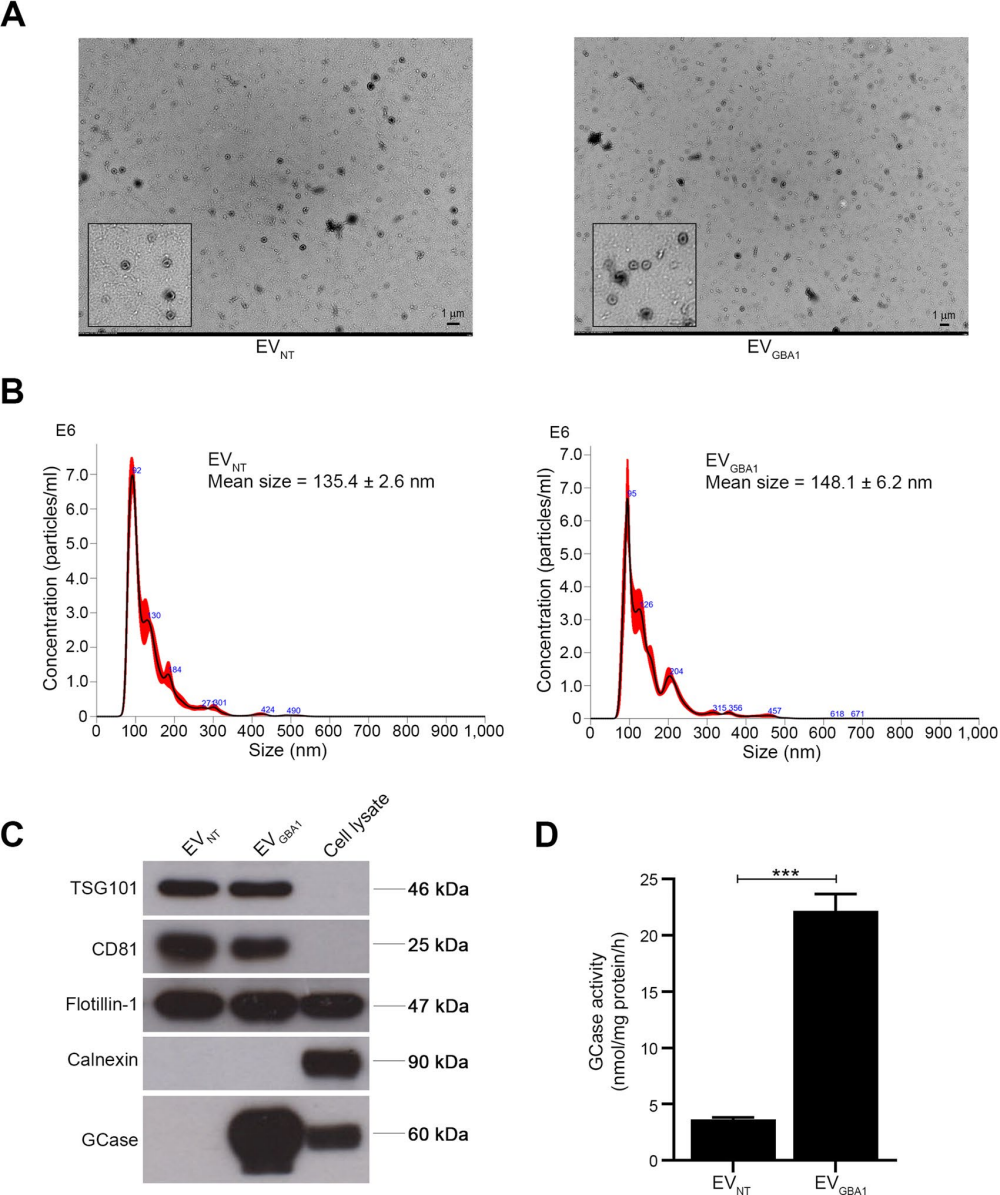
The characterization of EVs derived from HEK293T overexpressing *GBA1* cells. (**A**) EVs were isolated from cultured medium of HEK293T overexpressing *GBA1* cells through gradient ultracentrifugation. The EVs was visualized by transmission electron microscopy (TEM). Scale bar = 1 μm. (**B**) The size distribution of the isolated EVs was measured using Nanoparticle tracking analysis (NTA). (**C**) The protein markers of EVs (follitillin-1, TSG101 and CD-81), EVs negative marker (Calnexin) and the Gcase protein of isolated EVs and total cell lysates were analyzed using Western Blot analysis. (**D**) Gcase enzyme activity in isolated EVs were measured by enzymatic assay. Individual data relative to EV_NT_ are represented on bar chart with mean ± SEM, *n* = 3. ***p < 0.001 compared with EV_NT_ (unpaired student’s t-test)

To investigate the potential of extracellular vesicles (EVs) in carrying GCase, HEK293T-NT and HEK293T-*GBA1* cells were cultured in media supplemented with EV-depleted serum for 48 h. EVs were purified from the cultured medium via differential ultracentrifugation and characterized following the MISEV2018 guideline [21]. Negative-stain TEM revealed round-shaped vesicles with diameters of less than 200 nm (Fig. 2A). Further determination of EV size was conducted using nanoparticle tracking analysis (NTA), which revealed dimensions of 135.4 ± 2.6 nm for EVs derived from HEK293T-NT (EV_NT_) cells and 148.1 ± 6.2 nm for EVs derived from HEK293T-*GBA1* (EV_*GBA1*_) cells (Fig. 2B). The mean particle concentration was measured at 4.07 × 10^8^ particles/ml for EV_NT_ and 3.99 × 10^8^ particles/ml for EV_*GBA1*_. Additionally, the expression levels of EV protein markers were examined through western blotting. TSG101, CD81, and Flotillin-1 were found to be more abundantly expressed in EV fractions compared to cell lysate fractions. In contrast, calnexin was exclusively detectable in cell lysate fractions and absent from EV fractions (Fig. 2C). These results firmly established that the small vesicles isolated from both cell lines were indeed EVs. The expression of GCase in these EVs is depicted in bottom panel of Fig. 2C, where the GCase marker was prominently detectable only in EV_*GBA1*_ and not in EV_NT_. We further tested whether the expression of GCase also correlated with its enzymatic activity and found that GCase activity was significantly higher in EV_*GBA1*_ than in EV_NT_ (Fig. 2D). These results collectively confirm that EVs derived from HEK293T-*GBA1* cells carry the functional GCase enzyme.

### EVs containing GCase were internalized by THP-1 macrophage cells

**Fig. 3:**
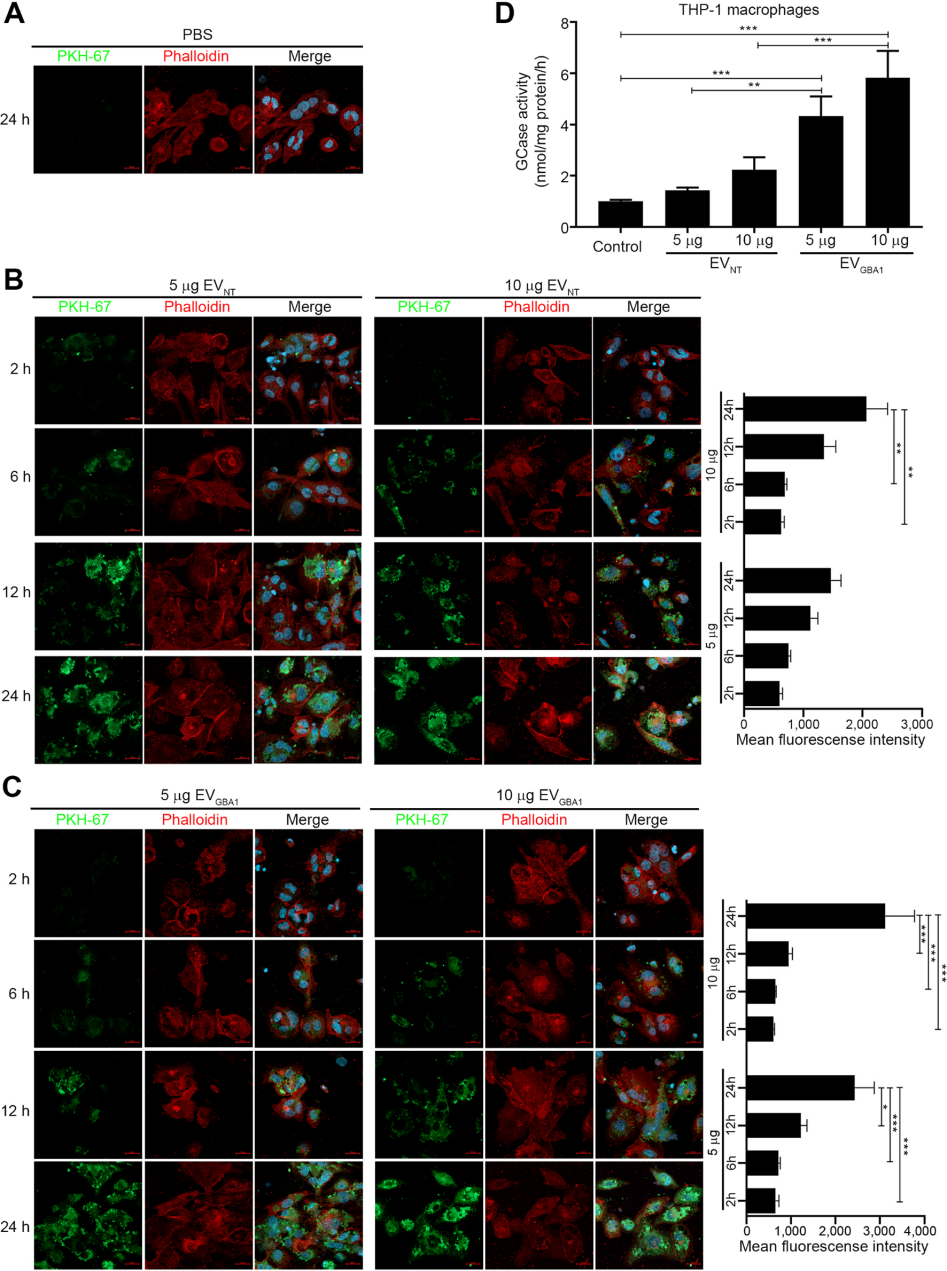
Uptake of HEK293T overexpressing *GBA1* cells-derived EVs into THP-1 macrophage cells. (**A-C**) The isolated EVs were labeled with PKH-67 dye and incubated in culture of THP-1 macrophage cells at 5 and 10 µg. After 2, 6, 12 and 24 h of incubation, the fluorescent signal was visualized by confocal microscopy. PKH-67 dye was used as an EVs marker (green). Phalloidin was use as actin filament marker (red). DAPI was used as a nuclear marker (blue). Scale bar = 20 μm. (**D**) Gcase enzyme activity in THP-1 macrophage cells were analyzed after incubation with isolated EVs at concentrations of 5 and 10 µg for 24 h. Individual data relative to control are represented on bar chart with mean ± SEM, *n* = 3. *p < 0.05, **p < 0.01, and ***p < 0.001 compared with control (one-way ANOVA with Dunett’s multiple comparison)

Macrophage is majorly affected in Gaucher disease and is often transformed into Gaucher cell that causes various organ abnormalities [7]. To assess the therapeutic potential of EVs derived from HEK293T-*GBA1* (EV_*GBA1*_) in macrophages, the following experiment was conducted. First, THP-1 monocytes were differentiated into macrophages. THP-1 macrophage cells were then incubated with 5–10 µg of green fluorochrome membrane dye (PKH-67)-labeled EVs for varying durations (2, 6, 12, and 24 h), and the EV uptake was visualized through confocal microscopy. PBS was used as a control. As illustrated in Fig. 3A-C, green fluorescence signal emitted by PKH-67-labeled EV_NT_ and EV_*GBA1*_ was evident in THP-1 macrophage cells, with a discernible increase in fluorescence intensity observed in a time- and dose-dependent manner. This outcome strongly suggests the capability of HEK293T stable cell-derived EVs to be internalized by THP-1 macrophage cells (*p* < 0.01). Additionally, GCase activity in THP-1 macrophage cells that had taken up EVs was quantified using an enzyme activity assay. After 24 h of EV incubation, GCase activity displayed a significant increase in THP-1 macrophage cells that had internalized EV_*GBA1*_ in comparison to both the PBS control and EV_NT_ (Fig. 3D). These findings indicate the retention of functional GCase enzyme within EV_*GBA1*_ that can be taken up by THP-1 macrophage cells.

### Enhancement of GCase activity in neuroblastoma cells by EV_*GBA1*_ delivery

**Fig. 4:**
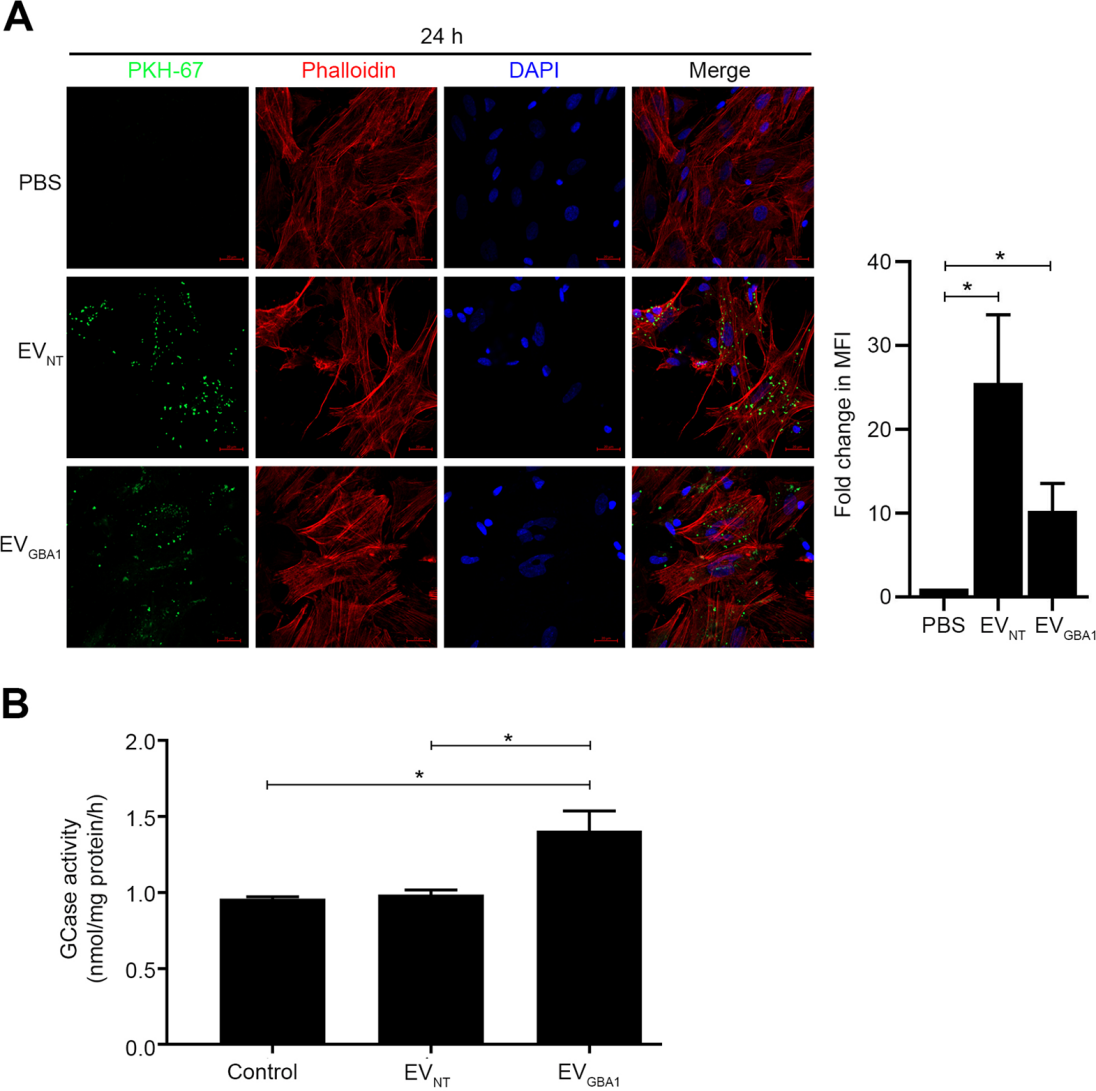
Uptake of HEK293T overexpressing *GBA1* cells-derived EVs into SH-SY5Y cells. (**A**) The isolated EVs were labeled with PKH-67 dye and incubated in culture of SH-SY5Y cells at 10 µg for 24 h. The fluorescent signal was visualized by confocal microscopy. PKH-67 dye was used as an EVs marker (green). Phalloidin was use as actin filament marker (red). DAPI was used as a nuclear marker (blue). Scale bar = 20. (**B**) Gcase activity in SH-SY5Y cells were analyzed after incubation with 10 µg of isolated EVs for 24 h. Individual data relative to EV_NT_ are represented on bar chart with mean ± SEM, *n* = 3. *p < 0.05 compared with EV_NT_ (unpaired student’s t-test)

Neuronal symptoms in type 2 and type 3 GD stem from reduced GCase activity within neuronal cell lysosomes [22]. To first assess the potential uptake of *GBA1*-carrying EVs by neuron-like cells, differentiated SH-SY5Y neuroblastoma cells were exposed to 10 µg of PKH-67-labeled EVs for a 24-hour duration. The findings demonstrated the incorporation of both EV_NT_ and EV_*GBA1*_ into SH-SY5Y cells (Fig. 4A). Importantly, GCase activity was significantly elevated in SH-SY5Y cells incubated with EV_*GBA1*_, while no significant change was observed in EV_NT_ compared to the PBS control (Fig. 4B). These findings indicate that EV_*GBA1*_ can be used to deliver functional GCase enzymes to neuron-like cells.

### GCase delivery via EVs to human differentiated macrophages from type 3 GD patient-derived iPSCs rescues enzymatic activity

**Fig. 5:**
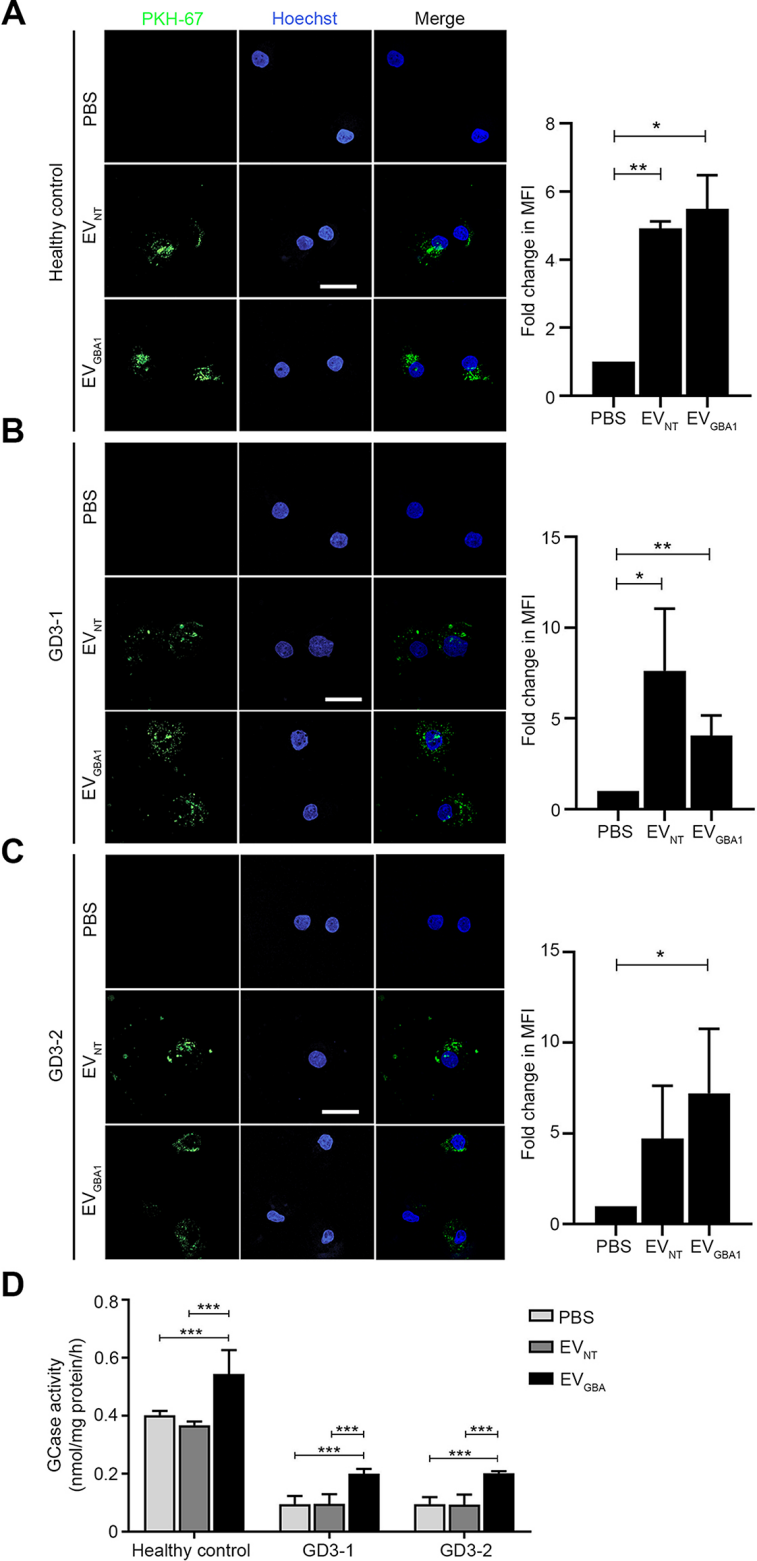
Uptake of HEK293T overexpressing *GBA1* cells-derived EVs into type 3 Gaucher iPSC-derived macrophage cells. (**A**) The healthy control-derived macrophage cells and (**B-C**) type 3 Gaucher (GD3-1 and GD3-2) iPSC-derived macrophage cells were incubated with 10 µg of PKH-67-labeled EVs for 24 h. The fluorescent signal was visualized by confocal microscopy. PKH-67 dye was used as an EVs marker (green). Hoechst was used as a nuclear marker. Scale bar = 10 μm. (**D**) Gcase activity was measured in GD3-2 cells after 24 h of incubation with 10 µg of isolated EVs. Individual data relative to EV_NT_ are represented on bar chart with mean ± SEM, *n* = 3. *p < 0.05, **p < 0.01, and ***p < 0.001 compared with EV_NT_ (unpaired student’s t-test)

The ultimate goal of using EVs containing functional GCase enzyme is to treat lysosomal storage disease defects at the cellular level. To this end, we first characterized the differentiation of macrophages derived from type 3 GD patient iPSCs (GD3-1/MUi030 and GD3-2/MUi031 cells) and from a healthy control iPSCs (MUi019-A). The surface markers (Fig. S2A) and morphology (Fig. S2B) confirmed successful terminal differentiation of macrophages. Next, EV_NT_ and EV_*GBA1*_ treatment showed noticeable fluorescence signal inside a healthy control, GD3-1 and GD3-2 iPSCs-derived type 3 GD patient macrophages, compared to PBS control (Fig. 5A-C). Importantly, EV_*GBA1*_ significantly improved the GCase enzyme activity in GD3-2 compared to PBS- and EV_NT_-treated groups (Fig. 5C). These findings demonstrate that EVs can be a vehicle to deliver and rescue GCase enzyme activity in type 3 GD patient-derived macrophages.

### EVs containing GCase were successfully internalized by neurons derived from both healthy individuals and patients with type 3 GD

**Fig. 6:**
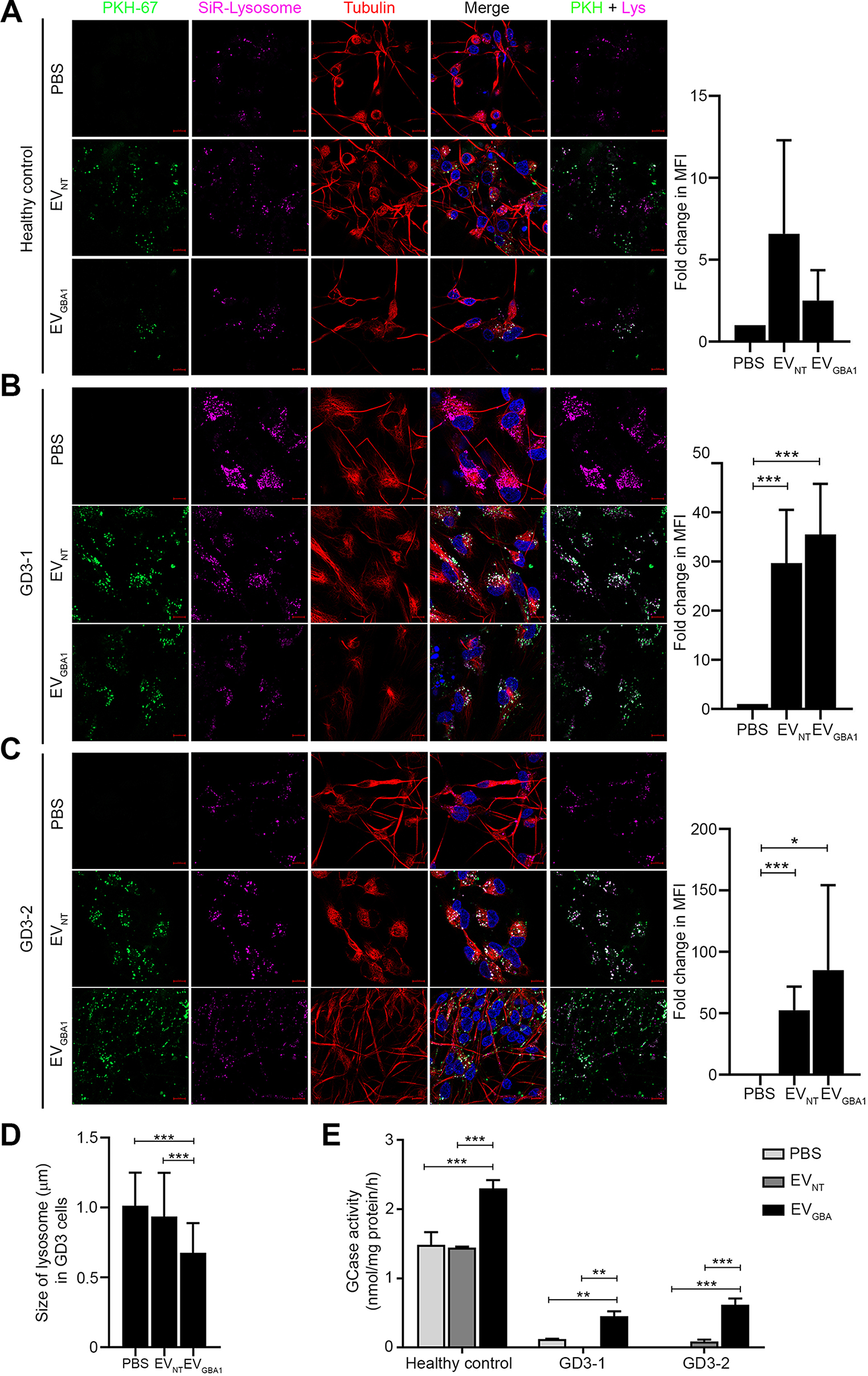
Uptake of HEK293T overexpressing *GBA1* cells-derived EVs into type 3 Gaucher iPSC-derived neuron cells. (**A**) The healthy control-derived neuron cells and (**B-C**) type 3 Gaucher (GD3-1 and GD3-2) iPSC-derived neuron cells were incubated with 10 µg of PKH-67-labeled EVs for 24 h. The fluorescent signal was visualized by confocal microscopy. PKH-67 dye was used as an EVs marker (green). SiR-Lysosome was used as a lysosome marker (pink). Tubulin marker was labeled with red color. Hoechst was used as a nuclear. Scale bar = 10 μm. (**D**) Size of lysosome in neurons derived from GD3-1 iPSCs were measured after incubation with 10 µg of isolated EVs for 24 h. (**E**) Gcase activity of macrophage derived from healthy control, GD3-1, and GD3-2 iPSCs were measured after incubation with 10 µg of isolated EVs for 24 h. Individual data relative to EV_NT_ are represented on bar chart with mean ± SEM, *n* = 3. *p < 0.05, **p < 0.01, and ***p < 0.001 compared with EV_NT_ (unpaired student’s t-test)

To test whether GCase-carrying EVs can reach defective lysosomes in neurons derived from type 3 GD patient cells, iPSCs were differentiated into NPCs and mature neurons, respectively. Both EV_NT_ and EV_*GBA1*_ were internalized by iPSC-derived neurons of all conditions (a healthy control, GD3-1 and GD3-2) (Fig. 6A-C). Moreover, the EV fluorescence signal colocalized with the lysosomal marker in iPSC-derived neuron cells, suggesting the successful uptake of GCase into neuronal lysosomes. To further confirm this, GCase activity was subsequently assessed in iPSC-derived neuronal cells after a 24-hour incubation with EVs. The findings indicate that GCase activity was notably lower in both GD3-1 and GD3-2 cells when exposed to PBS and EV_NT_, compared to that of a healthy control cells. Interestingly, EV_*GBA1*_ incubation appeared to reduce lysosomal size, clearly observed in GD3-1 cells (Fig. 6D). As expected, EV_*GBA1*_ incubation led to a significant increase in GCase activity in all iPSC-derived neuron conditions (Fig. 6E). These results collectively suggest that EVs containing functional GCase can be incorporated into the lysosomal compartment of neuronal cells, leading to increased GCase activity and rescued defective lysosomal phenotypes within GD patient-derived neuronal cells.

## Discussion

We have established a stable cell line that produces EVs containing functional lysosomal enzymes as an effective enzyme delivery approach to treat LSDs. Unlike other carriers/vehicles, EVs produced in human cells have intrinsic and specific mechanisms to enter living cells both in vitro and in vivo [23]. As a result, EVs hold promise for delivering protein payloads to targeted cells, including in the brain. Using this method, we demonstrated here that EVs released from *GBA1*-overexpressing cells successfully deliver GCase enzymes to THP-1 macrophage and SH-SY5Y neuronal cell lines and those human cells derived from neuropathic GD patient’s iPSCs, and successfully restore GCase enzyme activity in affected cell types. Our method may provide an alternative and more effective treatment option to manage GD, especially type 2 and type 3 with neurological complications.

Existing therapeutic options for GD patients are enzyme replacement therapy (ERT), substrate reduction therapy (SRT), and bone marrow transplantation (BMT), all of which are costly and have virtually failed to treat neurological symptoms of GD. The recombinant enzymes delivered by ERT method could not cross the BBB [24, 25]. Meanwhile, SRT trials with N-butyl deoxynojirimycin (Miglustat), which has the potential to cross the BBB, could not alleviate neurological symptoms [26]. Patients receiving BMT still showed neurological symptoms that progress after the transplantation [27]. Thus, it is in urgent need to find an alternative, effective therapeutic vehicle that (i) could cross the BBB and more importantly (ii) could rescue GCase function in neurons.

EVs are naturally released by all tissues for cell-to-cell communication, including between the brain cells and the rest of the body [28]. EVs produced from different tissue origins are thought to carry different license to allow for passing the BBB, thus making it intrinsically selective. Moreover, EVs can be produced in large quantity from engineered stable cell lines in a standard cell culture laboratory setting. Hence, EVs are well-equipped with potentials to scale up and to be capitalized for an enzyme or RNA delivery vehicle due to its stability after release from the cell and its ability to be stored long-term and easy for shipment [29]. In this study, we showed that isolated EVs from a commonly used HEK293T cell line contain functional GCase proteins that exhibit enzymatic activity. This is the first study to demonstrate the release of EVs containing functional GCase enzyme from *GBA1*-overexpressing HEK293T cells and, furthermore, showed the ability of these EVs to enter and enhance GCase activity in the human recipient cells, including neurons from patient’s iPSCs. Whether EVs produced from the HEK293T cells are able to effectively cross the BBB in vivo merits further investigation.

While most studies on enzyme replacement therapy aimed at establishing cells factories that serve as sustainable source of GCase, here we used stable cells that not only produced extremely high level of GCase protein but also released GCase retained in EVs, capable of entering target cells. Even if a small amount of GCase protein is packed into EVs and released compared to extrusion-based techniques, high concentration of EVs can be collected for treatment. Importantly, the concentration of EVs containing GCase used in this study is sufficient to reverse multiple cellular symptoms/pathologies of GD, including rescued enzymatic activity and reduced lysosomal size. The isolated fluorescently-tagged EVs were taken up by the recipient cells and were seen localized to endo-lysosomal compartments. Future investigations are needed to test whether internalized GCase via EVs can be sustained in the lysosomes for a long term.

In conclusion, this study provides compelling evidence that *GBA1*-overexpressing HEK293T cell-derived EVs, called EV_GBA_, have the capacity to carry functional GCase and enhance GCase activity in a variety of targeted cultured cells and cells derived from human patients. These findings offer promising avenues for further research into the therapeutic potential of lysosomal enzyme-carrying EVs in multiple types of LSDs, particularly in neuropathic GD, and broaden the applicability of EVs beyond their biological role as an intercellular communicator towards an effective enzyme delivery vehicle. Future studies may focus on optimizing EV-based therapies for specific cell targets affected in loss-of-function diseases, especially diseases affecting endo-lysosomes.

## Electronic supplementary material

Below is the link to the electronic supplementary material.

Supplementary Material 1: **Figure S1** The construction of GBA1 transfer plasmids used for producing GBA1 lentiviral vector. The vector contains cytomegalovirus (CMV) promoter, which drives the expression of GBA1 gene in all cell types. The transfer plasmid contains puromycin resistance gene, allowing the selection of cells containing GBA1 lentiviral vector

Supplementary Material 2: **Figure S2** Differentiation and characterization of human induced pluripotent stem cells (iPSC)-derived to macrophage, (Healthy control/MUi019), (GD3-1/MUi030), (GD3-2/MUi031). (A) Macrophage marker expression level in hiPSCs M-CSF-exposed macrophages. (B) Representative images of cell morphology hiPSC M-CSF-exposed macrophages. Scale bar represents 20 µm

## Data Availability

All data produced or examined throughout this study have been incorporated into this published article [along with its supplementary information files].

## Abbreviations

EVs: Extracellular vesicles
GD: Gaucher disease
GD3-1: iPSCs-derived type 3 GD patient 1 cells
GD3-2: iPSCs-derived type 3 GD patient 2 cells
GBA1: β-glucocerebrosidase gene
GCase: Glucocerebrosidase
LV-GBA1: Lentiviral Vector Carrying GBA1 Gene
HEK293T-GBA1: HEK293T Overexpressing GBA1 Gene
HEK293T-NT: Non-transduced HEK293T cells
EV_GBA1_: EVs derived from HEK293T-GBA1
EVNT: EVs derived from HEK293T-NT
LSDs: Lysosomal storage diseases
GlcCer: Glucosylceramide
ERT: Enzyme replacement therapy
SRT: Substrate reduction therapy
BMT: Bone marrow transplantation
RDP: Rabies glycoprotein-derived peptide
IgAh: Hinge region from IgA
VLPs: Virus-like nanoparticles
iPSCs: Induced pluripotent stem cells
HSCs: Hematopoietic stem cells
HEK293T: Human embryonic kidney cell line
THP-1: Human monocytic cell line
SH-SY5Y: Human neuroblastoma cell line
NPCs: Neural progenitor cells
FBS: Fetal bovine serum
PGK: Phosphoglycerate kinase
MOI: Multiplicity of infection
PMA: Phorbol 12-myristate 13-acetate
NTA: Nanoparticle Tracking Analysis
SDS: PAGE-Sodium dodecyl sulfate-polyacrylamide gel electrophoresis
TBST: Tris-buffered saline with Tween 20
PBS^++^: Ca^2+^-and Mg^2+^-containing PBS
4-MUG: 4-Methylumbelliferylβ-D-glucopyranoside substrates
4-MU: 4-Methylumbelliferone
