# Supplementary material for: Therapeutic delivery of recombinant glucocerebrosidase enzyme-containing extracellular vesicles to human cells from Gaucher disease patients
Source: Orphanet J Rare Dis. 2024 Oct 2;19:363. doi: 10.1186/s13023-024-03376-7 (PMC11445852; doi:10.1186/s13023-024-03376-7)
Supplement: Supplementary file 1 — Supplementary Material 1: Figure S1 The construction of GBA1 transfer plasmids used for producing GBA1 lentiviral vector. The vector contains cytomegalovirus (CMV) promoter, which drives the expression of GBA1 gene in all cell types. The transfer plasmid contains puromycin resistance gene, allowing the selection of cells containing GBA1 lentiviral vector [file 13023_2024_3376_MOESM1_ESM.docx]

**Supplementary**

**
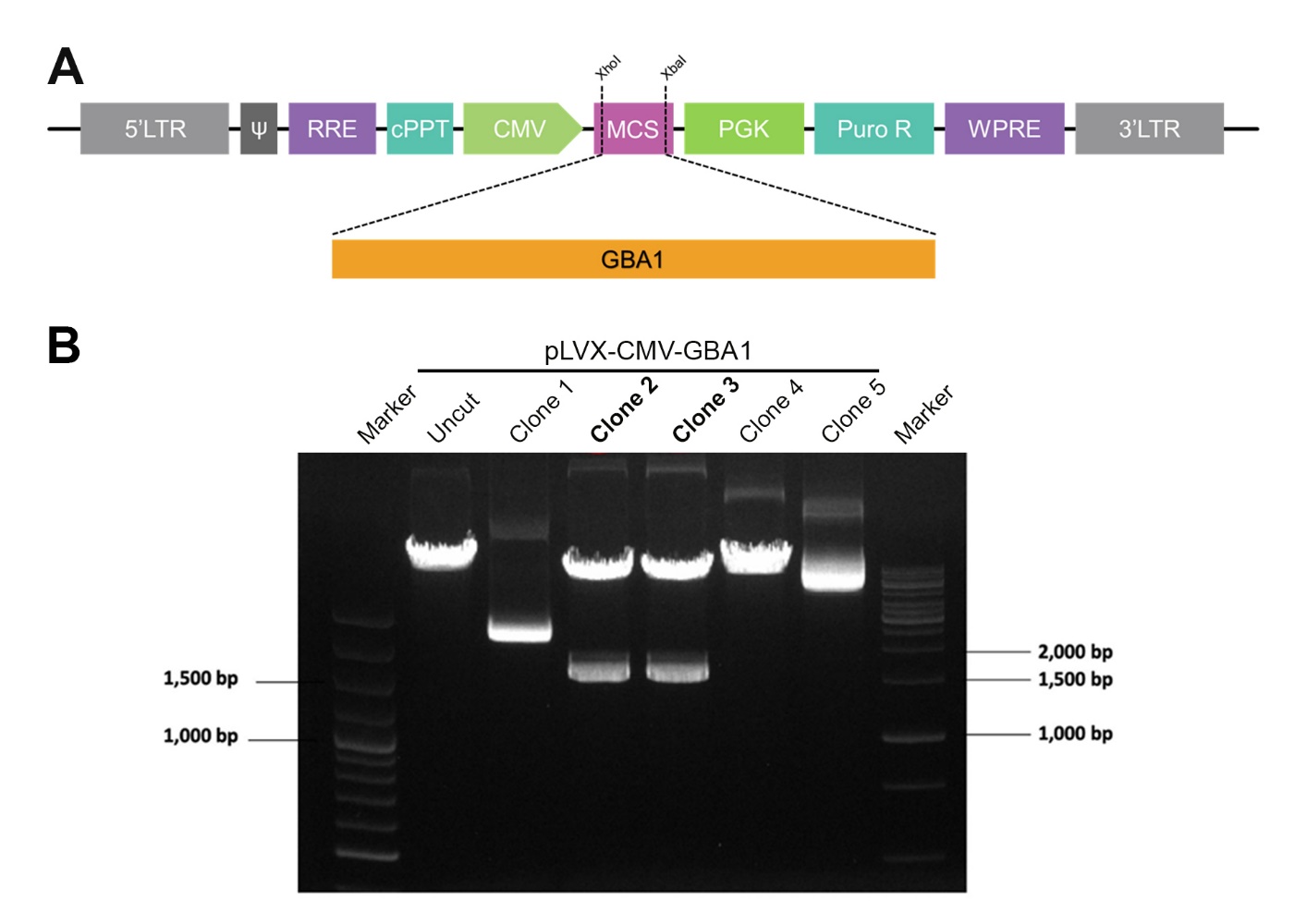
**

**Figure S1** The construction of *GBA1* transfer plasmids used for producing *GBA1* lentiviral vector. The vector contains cytomegalovirus (CMV) promoter, which drives the expression of *GBA1* gene in all cell types. The transfer plasmid contains puromycin resistance gene, allowing the selection of cells containing *GBA1* lentiviral vector.
