# Supplementary material for: Therapeutic delivery of recombinant glucocerebrosidase enzyme-containing extracellular vesicles to human cells from Gaucher disease patients
Source: Orphanet J Rare Dis. 2024 Oct 2;19:363. doi: 10.1186/s13023-024-03376-7 (PMC11445852; doi:10.1186/s13023-024-03376-7)
Supplement: Supplementary file 2 — Supplementary Material 2: Figure S2 Differentiation and characterization of human induced pluripotent stem cells (iPSC)-derived to macrophage, (Healthy control/MUi019), (GD3-1/MUi030), (GD3-2/MUi031). (A) Macrophage marker expression level in hiPSCs M-CSF-exposed macrophages. (B) Representative images of cell morphology hiPSC M-CSF-exposed macrophages. Scale bar represents 20 µm [file 13023_2024_3376_MOESM2_ESM.docx]

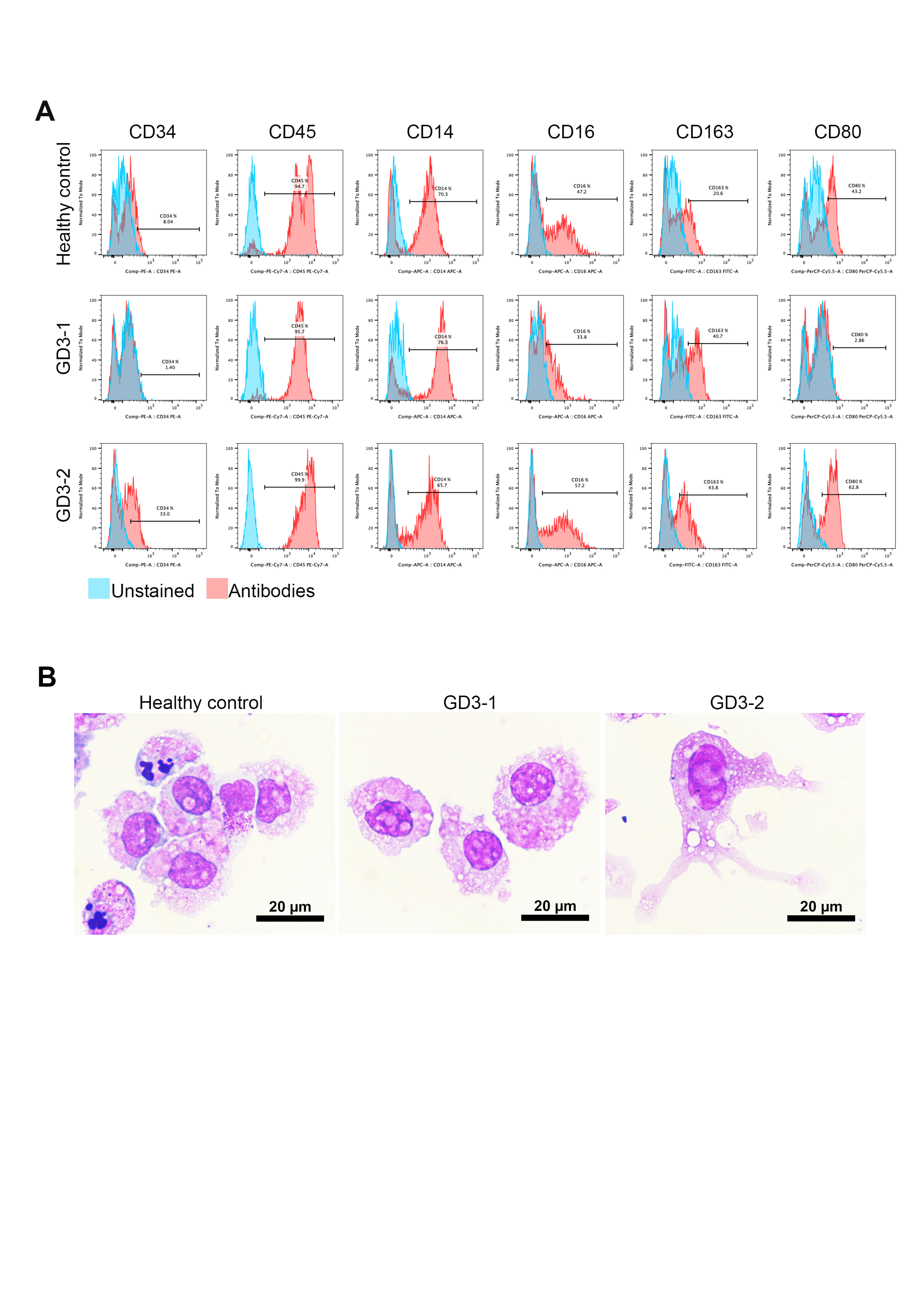


**Figure S2** Differentiation and characterization of human induced pluripotent stem cells (iPSC)-derived to macrophage, (Healthy control/MUi019), (GD3-1/MUi030), (GD3-2/MUi031). (A) Macrophage marker expression level in hiPSCs M-CSF-exposed macrophages. (B) Representative images of cell morphology hiPSC M-CSF-exposed macrophages. Scale bar represents 20 μm.
